# Comparison of outcomes of percutaneous coronary intervention for chronic total occlusion in patients with and without prior bypass grafting: A systematic review and meta-analysis

**DOI:** 10.12669/pjms.39.4.7483

**Published:** 2023

**Authors:** Dewei Wang, Keyu Chen, Tinglin Xiong, Ling He, Wei Ni, Haoyu Wang

**Affiliations:** 1 Dewei Wang, Department of Cardiology, Nanchong Central Hospital, Nanchong Central Hospital 97, Renmin South Road, Nanchong, Sichuan Province, 637000, China; 2 Keyu Chen Department of Outpatient, Nanchong Central Hospital, Nanchong Central Hospital 97, Renmin South Road, Nanchong, Sichuan Province, 637000, China; 3 Tinglin Xiong Department of Cardiology, Nanchong Central Hospital, Nanchong Central Hospital 97, Renmin South Road, Nanchong, Sichuan Province, 637000, China; 4 Ling He Department of Cardiology, Nanchong Central Hospital, Nanchong Central Hospital 97, Renmin South Road, Nanchong, Sichuan Province, 637000, China; 5 Wei Ni Department of Cardiology, Nanchong Central Hospital, Nanchong Central Hospital 97, Renmin South Road, Nanchong, Sichuan Province, 637000, China; 6 Haoyu Wang Department of Cardiology, Nanchong Central Hospital, Nanchong Central Hospital 97, Renmin South Road, Nanchong, Sichuan Province, 637000, China

**Keywords:** Chronic total occlusion, Bypass grafting, CABG, Percutaneous coronary intervention (PCI), Mortality

## Abstract

**Objective:**

This review assessed evidence on the impact of prior coronary artery bypass grafting (CABG) on outcomes of percutaneous coronary intervention (PCI) for chronic total occlusions (CTO).

**Methods:**

PubMed, CENTRAL, Embase, ScienceDirect, and Google Scholar databases were searched from 1st January 1980 up to 10th January 2022 for studies assessing outcomes of CTO-PCI in patients with and without prior-CABG.

**Results:**

Eight studies were included. The meta-analysis demonstrated significantly reduced odds of procedural success in patients with prior history of CABG (OR: 0.51 95% CI: 0.41, 0.64 I^2^=84% p<0.00001). There was a tendency of increased in-hospital mortality (OR: 1.72 95% CI: 0.97, 3.04 I^2^=26% p=0.06) and major adverse cardiac events (MACE) (OR: 1.30 95% CI: 0.99, 1.69 I^2^=0% p=0.05), along with a significantly increased risk of myocardial infarction (MI) (OR: 2.56 95% CI: 1.65, 3.97 I^2^=0% p<0.0001) and coronary perforation (OR: 1.52 95% CI: 1.03, 2.24 I^2^=70% p=0.04) in patients with history of CABG. There was no difference in the risk of stroke, pericardial tamponade, major bleeding, vascular access complications, and renal failure.

**Conclusion:**

Our results suggest that patients with prior history of CABG undergoing PCI for CTO have a 49% reduced chance of procedural success. Such patients are at an increased risk of in-hospital mortality, MACE, MI, and coronary perforation.

## INTRODUCTION

Chronic total occlusions (CTO) are amongst the most difficult coronary artery lesions to be treated with percutaneous coronary interventions (PCI).^1^ Previously, PCI for CTO lesions was associated with the high complication and reduced success rates.^2^ However, with continuous advances in CTO equipment and technology, clinical outcomes have greatly improved.^3^ A recent trial has demonstrated that CTO-PCI is indeed feasible with good success rates and there is no difference in the risk of major adverse cardiovascular events (MACE) between CTO and non-CTO-PCI.^4^ Research has also shown that successful PCI for CTO lesions leads to improved quality of life, better left ventricular function, improved survival, and a decreased risk of coronary artery bypass grafting (CABG).^5^,^6^

Indeed, the use of PCI for CTO has increased significantly in the past decade with a corresponding increase in procedural success.^7^ Evidence from randomized controlled trials suggests that in comparison to PCI, CABG results in significantly better long-term outcomes in complex coronary artery diseases.^8^,^9^ However, it is also known that CABG itself precipitates atherosclerosis in the native coronary arteries.^10^ CABG alters the blood flow and induces stasis, negative remodeling, and calcifications leading to accelerated development of atherosclerotic lesions in native coronary arteries proximal to the grafted site.^11^ Indeed, prediction models for CTO-PCI have reported a prior history of CABG to be a risk factor for procedural failure.^12^

Several studies have attempted to compare outcomes of CTO-PCI between patients with and without a history of prior CABG but with conflicting results. While some^13^ report worse outcomes in patients with prior CABG others^14^ suggest no such difference. Two meta-analysis studies have been conducted on this topic.^15^,^16^ While one review^16^ could only include studies published up to 2019, another recent review^15^ included studies with overlapping data reporting outcomes from the same institute or PCI registries thereby resulting in biased reporting. Furthermore, more recently published studies^14^,^17^ were not included in both these reviews. Therefore, to overcome the limitations of past reviews, we designed the current study to compare in-hospital and long-term outcomes of CTO-PCI in patients with and without prior CABG.

## METHODS

This review was reported based on the PRISMA recommendations^18^ and was pre-registered on the PROSPERO database (CRD42022299439).

### Literature search:

PubMed, CENTRAL, Embase, ScienceDirect, Google Scholar were searched for English language studies from 1^st^ January 1980 up to 10^th^ January 2022. The search terms were: “chronic total occlusion”, “CTO”, “percutaneous coronary intervention”, “PCI”, “coronary artery bypass”, and “CABG” (Supplementary Table-I). The search results were consolidated, deduplicated, and screened by title and abstracts by two reviewers separately. Articles of interest to the review were selected and downloaded for full-text analysis. They were cross-checked against the inclusion criteria for final selection. Disagreements between the two reviewers were cleared in consultation with another reviewer.

### Inclusion criteria on PECO format was:

- Population: patients undergoing PCI for CTO.
- Exposure: Patients with prior history of CABG
- Comparison: patients without any prior history of CABG.
- Outcomes: success rates, procedural complications, MACE, mortality, stroke, myocardial infarction (MI), bleeding, cardiac tamponade, coronary perforation, renal failure, or target vessel revascularization (TVR).

### Exclusion Criteria:

We excluded the following:

- Non-comparative studies,
- Those not reporting required outcomes,
- Editorials, review articles and with duplicate data.

### Data extraction and quality assessment:

The reviewers sourced author details, study year and type, study location and database, sample size, demographic details, smokers, comorbidities (Hypertension, diabetes mellitus, renal disease, dyslipidemia), previous MI or PCI, target vessel, the approach of PCI, prior failed attempts, Japanese CTO score (J-CTO), vessel calcification, blunt stump, procedural time, contrast volume, study outcomes, and follow-up.

### Procedural success as the primary outcome of our review:

It was defined as residual stenosis <30% and TIMI flow grade ≥3 without any MACE in all studies, except for one wherein <50% residual stenosis was considered as a successful procedure. Other outcomes of interest were all-cause mortality, MACE, MI, stroke, coronary perforation, pericardial tamponade, major bleeding, vascular access complications, renal failure, and TVR. Based on the follow-up duration, outcomes were separated into in-hospital and late outcomes (>6 months of follow-up). We assessed the risk of bias using the Newcastle-Ottawa scale (NOS).^19^

### Statistical analysis:

We used “Review Manager” (RevMan, version 5.3; Nordic Cochrane Centre [Cochrane Collaboration], Copenhagen, Denmark; 2014) for this study. Data was combined to compute odds ratios (OR) and 95% confidence intervals (CI). We also sourced adjusted hazard ratios (HR) of long-term outcomes and combined them to compute the total effect size. All meta-analyses were conducted using the random-effects model.

We assessed inter-study heterogeneity using the I^2^ statistic. We assessed publication bias for the primary outcome by visual inspection of funnel plots. A sensitivity analysis was conducted for procedural success and all-cause mortality to assess if any study had an exaggerated effect on the pooled estimate.

## RESULTS

### Search:

**Fig.1:**
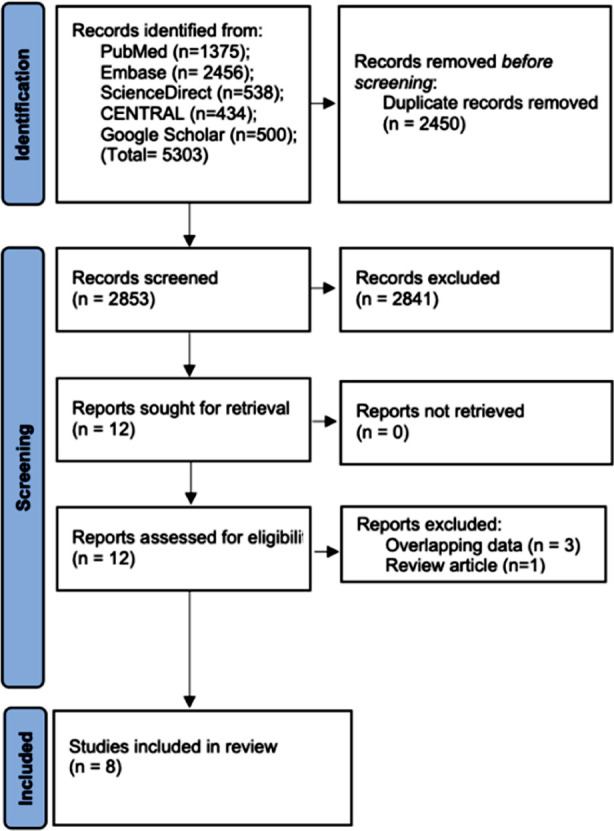
Study flow-chart.

2853 unique articles were found (Fig.1). Full-texts of these 12 studies was reviewed. Three^20^-^22^ were found to report overlapping data while one^23^ was a review article. Finally, eight studies were included in our review.^13^,^14^,^17^,^24^-^28^

Baseline details are presented in Tables-I and II. All were retrospective observational studies. A total of 6182 PCI procedures for CTO were conducted after CABG in the included studies and these were compared with a control group of 25445 CTO-PCI procedures. Four studies reported only in-hospital data while the other four studies also reported long-term data. The NOS score of the studies ranged from six to eight. The majority of the studies lacked baseline matching of study cohorts.

**Table-I:** Details of included studies

Study	Location	Database	Groups	Sample size	Mean/ Median age (years)	Male gender (%)	DM (%)	HT (%)	DL (%)	Smoking (%)	Renal disease (%)	Previous MI (%)	Previous PCI (%)	Follow-up	NOS score
Micheal 2013[24]	USA	3 USA centers (2006-2011)	pCABG nCABG	508 855	67.7 63.3	86.2 84.2	44.3 36.8	92.6 87.2	96 92.6	8 4.5	NR	44.9 39.8	43.4 40.8	IH	6
Teramoto 2014[25]	Japan	Toyohashi Heart Center (1999-2011)	pCABG nCABG	153* 1139	68.2 66	82 82	42 37	59 61	35 37	18 25	10 6	NR	NR	IH	6
Toma 2016[26]	Germany	University Heart Center (2005-2013)	pCABG nCABG	292 1710	68 65	88 83	39 28	90 81	91 85	7 22	27 19	48 21	23 14	2.6 years	8
Azzalini 2018[27]	Multicentric	Seven different centers (2009-2017)	pCABG nCABG	401 1657	69.2 64.3	92 87	48 35	87 74	91 78	12 31	26 18	56 43	73 58	2 years	6
Budassi 2020[28]	Multicentric	RECHARGE registry (2014-2015)	pCABG nCABG	217 1035	68.5 64.9	86.2 85.5	31.3 25.5	72.4 59.3	78.3 64.7	7.4 24.6	16.6 10.5	51.2 36.6	62.5 55.9	IH	6
Nikolakopoulos 2020[13]	Multicentric	PROGRESS-CTO registry (2012-2019)	pCABG nCABG	1074 498	67 63	87 81	52 40	94 90	94 82	17 27	NR	61 46	75 60	1 year	6
Hernandez-Suarez 2021[17]	Multicentric	LATAM CTO registry (2008-2020)	pCABG nCABG	251 1411	67 64	80.5 78.2	48.2 35.8	93.6 86.5	83.7 70.6	17.3 18.1	3.8 3.2	48.8 40.2	57.6 49.3	IH	8
Shoaib 2021[14]	UK	British Cardiovascular Intervention Society database (2007-2014)	pCABG nCABG	3233 16848	68 63	86 76	32 21	69 61	69 65	63 64	1.49 0.81	59 39	50 33	1 year	8

pCABG; prior coronary artery bypass grafting; nCABG, no prior coronary artery bypass grafting; DM, diabetes mellitus; HT, hypertension; DL, dyslipidemia; MI, myocardial infarction; PCI, percutaneous coronary intervention; NOS, Newcastle Ottawa scale; IH, in-hospital

* no of PCI procedures in pCABG and nCABG group were 206 and 1431 respectively.

**Table-II:** CTO characteristics of the included studies

Study	Groups	Target vessel-RCA (%)	Target vessel-LAD (%)	Target vessel-LCX (%)	Prior failed attempts (%)	J-CTO score	Calcification (%)	Blunt stump (%)	AWE (%)	ADR (%)	Retrograde approach (%)	Procedure time (mins)	Contrast volume (ml)
Micheal 2013[24]	pCABG nCABG	56.2 54.7	14.2 25	27.4 20.1	13 16.6	NR	NR	NR	94.2 97.5	29.4 28.7	46.7 27.1	125± 65 106± 58	296± 156 293± 160
Teramoto 2014[25]	pCABG nCABG	45 43	22 34	31 22	NR	NR	53 33	NR	NR	NR	NR	210.3± 98.1 165.6± 77.3	NR
Toma 2016[26]	pCABG nCABG	44 47	15 30	37 23	NR	NR	71 53	NR	NR	NR	42 21	NR	371± 170 311± 152
Azzalini 2018[27]	pCABG nCABG	53 49	21 31	26 20	NR	2.3± 1.2 1.7± 1.2	59 40	53 40	40 62	20 15	40 22	157± 80 115± 64	326± 129 309± 136
Budassi 2020[28]	pCABG nCABG	67.3 59.1	8.3 26.3	22.6 14.6	21.7 21.3	2.9± 1.2 2.1± 1.2	77.4 54.2	62.2 47.2	57.1 84.3	23.5 23.2	58.5 28.4	116.2± 54.2 92.6± 50	312.2± 159.9 252.2± 125.2
Nikolakopoulos 2020[13]	pCABG nCABG	56 54	17 30	25 16	18 17	2.9± 1.1 2.2± 1.3	62 36	57 45	79 88	30 25	47 28	154[110-214] 106[71-155]	250 [175-340] 225[160-300]
Hernandez-Suarez 2021[17]	pCABG nCABG	47.7 42.1	15.6 37.2	33.7 20.5	20.2 14.7	2.5± 1.2 2.1± 1.2	62.3 43.9	56.5 49.1	80.2 92.8	6.2 2.1	13.7 5.2	NR	271.5± 134 245.5±113.7
Shoaib 2021[14]	pCABG nCABG	43 53	17 40	28 23	NR	NR	NR	NR	NR	NR	NR	NR	NR

RCA, right coronary artery; Left anterior descending artery; LCX, Left circumflex artery; AWE, Antegrade wire escalation; ADR, antegrade dissection and reentry; Japanese chronic total occlusion registry.

### Procedural success:

**Fig.2:**
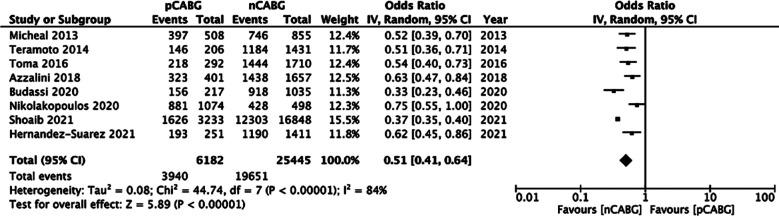
Meta-analysis of procedural success between prior CABG (pCABG) and CABG-naïve patients (nCABG).

**Supplementary Fig.1:**
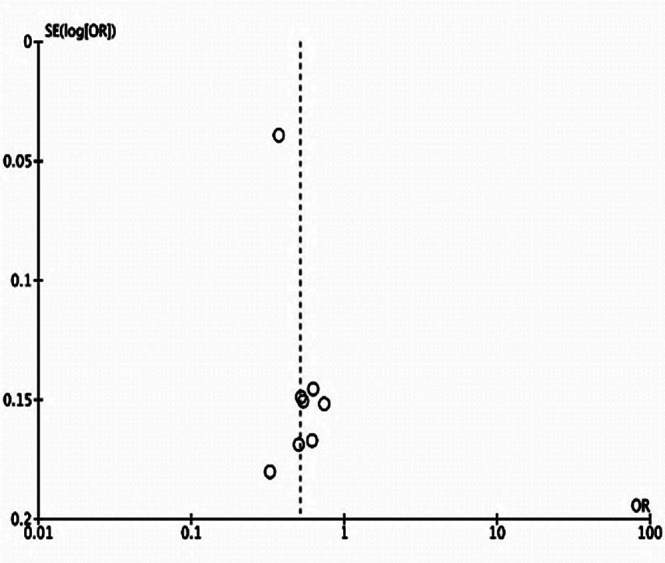
Forest plot for the meta-analysis of procedural success.

The meta-analysis showed significantly reduced odds of procedural success in patients with prior history of CABG (OR: 0.51 95% CI: 0.41, 0.64 I^2^=84% p<0.00001) (Fig.2). There was no publication bias (Supplementary Fig.1). The results did not differ on sensitivity analysis.

### In-hospital mortality:

**Fig.3:**
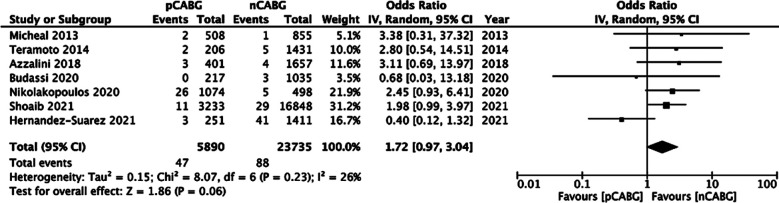
Meta-analysis of in-hospital all-cause mortality between prior CABG (pCABG) and CABG-naïve patients (nCABG).

Pooled analysis indicated a tendency of increased mortality in patients with prior history of CABG, but the results were not statistically significant (OR: 1.72 95% CI: 0.97, 3.04 I^2^=26% p=0.06) (Fig.3). On the exclusion of the study of Hernandez-Suarez et al,^17^ the results indicated a statistically significant increased risk of mortality in patients with a history of CABG (OR: 2.24 95% CI: 1.38, 3.64 I^2^=0% p=0.001).

### Complications:

**Supplementary Fig.2:**
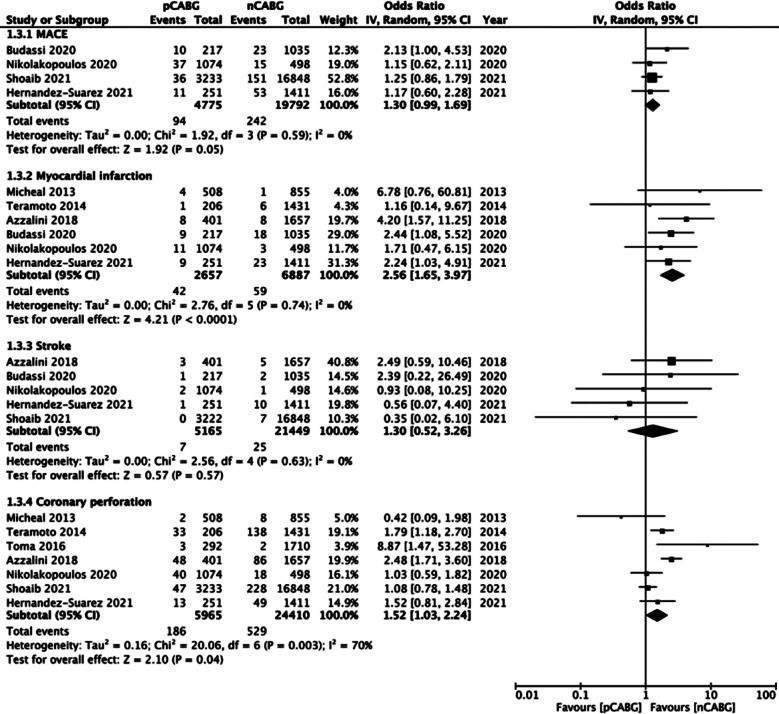
Meta-analysis of complications (MACE, MI, stroke, coronary perforation) between prior CABG (pCABG) and CABG-naïve patients (nCABG).

Based on the available data, our meta-analysis revealed a tendency of increased risk of MACE (OR: 1.30 95% CI: 0.99, 1.69 I^2^=0% p=0.05) along with a statistically significant increased risk of myocardial infarction (OR: 2.56 95% CI: 1.65, 3.97 I^2^=0% p<0.0001) and coronary perforation (OR: 1.52 95% CI: 1.03, 2.24 I^2^=70% p=0.04) in patients with history of CABG, but no difference in the risk of stroke (OR: 1.30 95% CI: 0.52, 3.26 I^2^=0% p=0.57) between the two groups (Supplementary Fig.2).

**Supplementary Fig.3:**
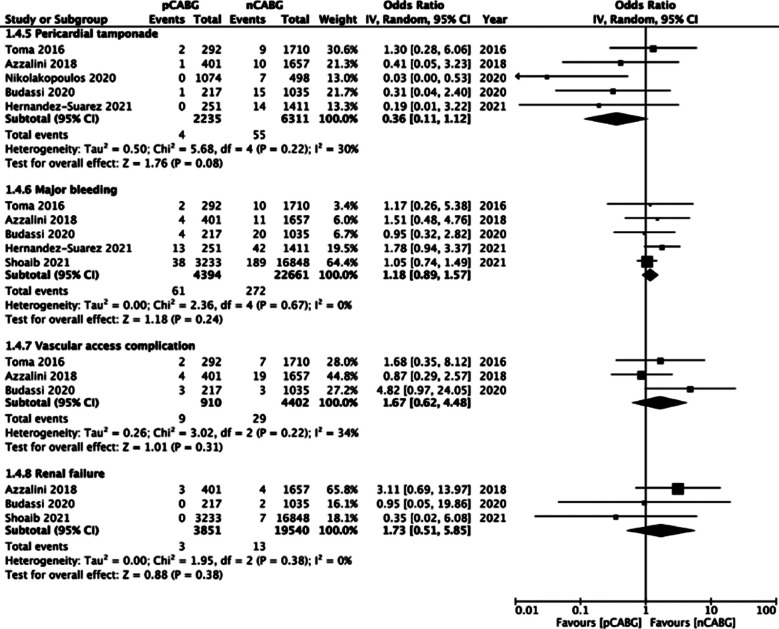
Meta-analysis of complications (pericardial tamponade, major bleeding, vascular access complications, renal failure) between prior CABG (pCABG) and CABG-naïve patients (nCABG).

However, meta-analysis revealed no difference in the risk of pericardial tamponade (OR: 0.36 95% CI: 0.11, 1.12 I^2^=30% p=0.08), major bleeding (OR: 1.18 95% CI: 0.89, 1.57 I^2^=0% p=0.24), vascular access complications (OR: 1.67 95% CI: 0.62, 4.48 I^2^=34% p=0.31) and renal failure (OR: 1.73 95% CI: 0.51, 5.85 I^2^=0% p=0.38) between patients with prior history of CABG and CABG-naive patients (Supplementary Fig.3).

### Long-term outcomes:

**Supplementary Fig.4:**
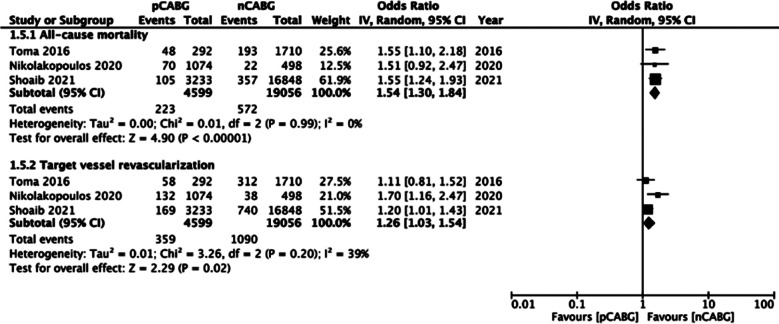
Meta-analysis of long-term all-cause mortality and TVR between prior CABG (pCABG) and CABG-naïve patients (nCABG).

Limited long-term outcome data were available and a pooled analysis was possible only for mortality and TVR. Meta-analysis revealed an increased risk of all-cause mortality in patients with prior history of CABG on long-term follow-up (OR: 1.54 95% CI: 1.30, 1.84 I^2^=0% p<0.00001). Similarly, patients with prior CABG had a higher odds of TVR as compared to the CABG-naïve group (OR: 1.26 95% CI: 1.03, 1.54 I^2^=39% p=0.02) (Supplementary Fig.4).

**Supplementary Fig.5:**
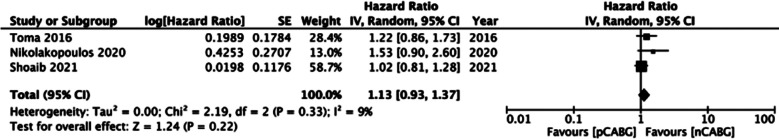
Meta-analysis of long-term adjusted mortality between prior CABG (pCABG) and CABG-naïve patients (nCABG).

Adjusted data from a minimum of three studies were available only for all-cause mortality. Quantitative analysis showed no statistically significant difference in long-term mortality (HR: 1.13 95% CI: 0.93, 1.37 I^2^=9% p=0.22) (Supplementary Fig.5).

## DISCUSSION

Historically, limited number of CTO patients were treated by PCI.^29^ However, technological improvements has changed the clinical scenario. Research shows that compared to medical therapy, PCI for CTO resulted in lower long-term mortality with no difference in the incidence of major complications.^30^ Nevertheless, the success rates achieved by CTO-PCI have generally been approximately 15% lower as compared to PCI for non-CTO lesions.^30^,^31^ Lower success rates have in turn been related with adverse long-term outcomes and reduced overall survival.^32^ Several studies have shown that prior CABG could be a risk factor for lower success rates in CTO-PCI.^33^,^34^

To better elucidate the influence of prior CABG on CTO-PCI outcomes, we conducted a meta-analysis. We noted that in patients with prior CABG the odds of procedural success are significantly reduced. Our results are similar to the prior meta-analysis of Liu et al.^16^ However, the more recent review of Shi et al^15^ did not analyze success rates. Lower procedural success with prior CABG could be attributable to several reasons. Firstly, the difference in the baseline demographic and patient characteristics are important contributors to PCI success.

It can be seen that in the majority of the included studies, patients with prior CABG were older, more male, had a higher incidence of comorbidities like diabetes, hypertension, dyslipidemia, renal disease along with the greater frequency of prior MI and PCI. Prior CABG patients also had complex lesions which makes the procedure challenging. Secondly, the association between prior CABG and aggressive development of atherosclerosis in native coronary arteries is quite well established.^10^ Research suggests that CTO lesions in surgically revascularized patients have greater calcification, moderate negative remodeling, and excessive prevalence of blunt stumps compared to CABG naïve patients.^11^

The escalated risk of atherosclerosis after CABG has been attributed to abnormal flow patterns resulting in altered shear stress.^35^ The development of blood stasis with such atypical blood flow could contribute to the higher calcification rates found in native coronary arteries of post-CABG patients.^11^ Such negative angiographic features could therefore impact the success rates of CTO-PCI. Thirdly, distortion and displacement of native vessel anatomy by prior CABG could also hinder CTO crossing attempts leading to higher failure rates.^16^ Furthermore, a larger incidence of complications in prior CABG patients could reduce the procedural success.

Indeed, our meta-analysis revealed that there was an increased tendency of in-hospital mortality and MACE in patients with a history of CABG, but without statistical difference. This could be due to the low number of events in the included studies. Secondly, there was increased risk of MI and coronary perforation in patients with a history of CABG, but no difference in other complications. These results are similar to Liu et al.^16^ However, Shi et al^15^ in their meta-analysis have also noted the reduced incidence of cardiac tamponade and higher rates of contrast-induced nephropathy in patients with prior CABG.

It is pertinent to mention that the studies included in our review and that of Shi et al^15^ are not the same. Two of the studies^21^,^22^ included by Shi et al^15^ had overlapping data with Azzalini et al^27^and to evaluate the role of the Registry of CrossBoss and Hybrid procedures in France, the Netherlands, Belgium, and United Kingdom (RECHARGE and Nikolakopoulos et al^13^ and hence were excluded from our meta-analysis. Including the same patients twice in a pooled analysis can exaggerate the effect of the intervention thereby generating false results. We also added two recently published studies with large sample sizes thus providing updated evidence.

The higher incidence of coronary perforation in prior CABG patients could be attributable to the aggressive techniques used in such patients. The baseline data of the included studies show that prior CABG patients frequently underwent the retrograde and dissection re-entry approach as compared to CABG-naive patients. The retrograde approach associated collateral channel damage and the requirement of aggressive balloon dilation in the severely atherosclerotic vessels of CABG patients could also increase the risk of coronary perforations.^27^

Our review is the first to pool evidence on long-term outcomes of CTO-PCI between prior CABG and CABG naïve patients. Acknowledging the fact that long-term data is currently scarce, we noted higher mortality rates in prior CABG patients on the meta-analysis of crude data but the results were no longer significant on analysis of adjusted data. This indicates that the baseline differences in the prior CABG group are the primary drivers of poor survival. Crude data on TVR did indicate a higher need for revascularization in prior CABG patients, but we were unable to pool adjusted outcomes for TVR due to unavailability of data from the included studies.

### Limitations:

Firstly, the current evidence is derived from only retrospective observational studies which have an inherent risk of bias. Secondly, PCI in the included studies was performed over a large period ranging from 1999 to 2020. The current results do not take into account the technological developments and technique improvements for CTO-PCI occurring over such a long time duration. Also, the procedures were carried out at different centers worldwide by operators of varying experience.

PCI for CTO is a highly skilled procedure and success rates are directly proportional to operator and hospital experience.^36^ Thirdly, our meta-analysis was a study level and not a patient-level meta-analysis. The latter would have provided better evidence. Lastly, maximum outcomes pooled in our study were from crude and not adjusted data. The majority of the included studies did not carry out baseline matching of the study groups and failed to report multivariable-adjusted data.

### Future directions:

Further prospective studies with long-term follow-up are needed to generate better quality evidence on the effect of prior CABG on outcomes of CTO-PCI. Future studies should carry out baseline matching of patient characteristics and reported multi-variable adjusted data for better interpretation of the available evidence.

## CONCLUSION

Patients with prior history of CABG undergoing PCI for CTO have a 49% reduced chance of procedural success. Such patients are at an increased risk of in-hospital mortality, MACE, MI, and coronary perforation. The reduced success rates and higher complications in prior CABG patients are probably related to the unfavourable patient demographics, higher comorbidities, and increased complexity of CTO lesions.

### Authors’ contributions:

**DW:** Conceived and designed the study.

**KC, TX, LH, WN and HW:** Collected the data and performed the analysis.

**XW:** was involved in the writing of the manuscript and is responsible for the integrity of the study.

All authors have read and approved the final manuscript.
